# Compositional Acclimation Can Lessen Tropical Forest Change in Response to Increasing Lightning Frequency: Insights From Simulation Modeling

**DOI:** 10.1111/gcb.70635

**Published:** 2025-12-08

**Authors:** David Medvigy, Evan M. Gora, Stephen P. Yanoviak

**Affiliations:** ^1^ Department of Biological Sciences University of Notre Dame Notre Dame Indiana USA; ^2^ Cary Institute of Ecosystem Studies Millbrook New York USA; ^3^ Smithsonian Tropical Research Institute Balboa Panama; ^4^ Department of Biology University of Louisville Louisville Kentucky USA

**Keywords:** climate change, compositional acclimation, forest dynamics model, lightning, mortality, tropical forest dynamics

## Abstract

Lightning frequency in tropical forests has been increasing for decades and lightning is a major agent of forest biomass mortality, but the implications of increased lightning frequency are unclear. Here, we provide a species‐ and spatially explicit implementation of lightning in a mechanistic forest dynamics model. We evaluated the model's ability to reproduce current‐day observations in a Panamanian tropical forest, and the sensitivity of model outputs to plausible changes in lightning frequency. The lightning‐enabled model simulated aboveground biomass (AGB), carbon flux, and stem densities that were consistent with observations. As expected, AGB declined with increasing lightning frequency. However, the magnitude of AGB decline was greatly reduced when trees were assigned empirically derived, species‐specific lightning tolerances. Changes in species composition weakened the sensitivity of AGB to increasing lightning: the AGB of a small number of large‐statured, lightning‐tolerant species increased with increasing lightning frequency. In addition, the effect of lightning on AGB tended to saturate at high lightning frequencies because of the combined effect of changes in size structure and composition. Specifically, the number of large, lightning‐susceptible trees was relatively small at high lightning frequencies. Overall, this study shows that an empirically informed representation of lightning captures the contemporary effects of lightning on forests, indicates that changes in lightning frequency will change forest AGB, species composition, and size structure, and shows that forests can partially acclimate to higher lightning frequency through changes in composition. Thus, more widespread inclusion of the lightning into global ecosystem models would be an important step toward improving simulations of forest responses to global change.

## Graphical abstract

Lightning is a major agent of forest tree mortality. In this study, we investigated how changes in lightning frequency affect forest biomass. Our approach was to implement lightning into a forest dynamics model. The probability of lightning‐induced mortality varied by species according to field observations. We found that increases in lightning frequency reduced forest biomass, but only by a modest amount. Several large‐statured, lightning‐tolerant species increased in biomass as lightning frequency increased. We conclude that forest biomass can partially acclimate to increases in lightning frequency through changes in species composition.

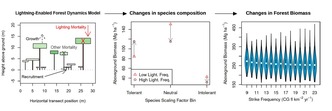

## 1 Introduction

Tropical intact forests sequestered about 14% of anthropogenic carbon emissions since the 1990s (Pan et al. 2024). However, changes in disturbance regimes are transforming these forests, leading to a reduction in carbon sink strength (Pugh et al. 2019; Hubau et al. 2020; Yang et al. 2021; Pan et al. 2024), compositional change (Feeley et al. 2011; Fauset et al. 2012; Esquivel‐Muelbert et al. 2019), and changes in forest structure (Gora and Esquivel‐Muelbert 2021; Needham et al. 2022). Mechanistic forest dynamics models are key tools for understanding the interactions between forests and climate (Bugmann and Seidl 2022), but they have struggled to simulate disturbances and disturbance‐related mortality (Koch et al. 2021). Until recently, none of these models have included lightning (Krause et al. 2025), which is a key agent of tropical tree mortality and biomass carbon turnover (Furtado 1935; Anderson 1964; Magnusson et al. 1996; Gora and Yanoviak 2020; Yanoviak et al. 2020; Gora, Burchfield, et al. 2020; Veraverbeke et al. 2025).

Lightning strikes hit tropical forests 35–67 million times per year (Gora, Burchfield, et al. 2020). Studies of Amazonian forests have reported that higher lightning strike frequency is correlated with shorter maximum tree heights (Gorgens et al. 2021) and lower taxonomic diversity among large trees > 70 cm in diameter (de Lima et al. 2023). Field surveys of lightning strike sites in Panama and Peru indicate that lightning kills 5–6 trees per strike (Gora and Yanoviak 2020; Gora and Esquivel‐Muelbert 2021), and post hoc assessment of forest gaps in Brazil and Malaysia has reported even larger numbers of mortalities per strike (Anderson 1964; Magnusson et al. 1996). However, direct observations of lightning‐associated biomass mortality are very limited. At the only site with such observations, an old‐growth lowland tropical forest at Barro Colorado Island (BCI) in Panama, lightning was estimated to cause 40%–50% of mortality among trees > 60 cm in diameter and contribute at least 16% of total biomass mortality in this forest (Gora and Esquivel‐Muelbert 2021). This estimate includes only the direct effect of lightning on trees and does not include fire. The observed effect of lightning on large trees is particularly notable because large trees account for approximately half of tropical forest aboveground biomass (AGB) carbon storage, productivity, and mortality (Slik et al. 2013; Meakem et al. 2018; Lutz et al. 2018; Ali et al. 2019).

Analysis scaling up these results and accounting for spatial variation in tropical forest lightning strike frequency (Gora, Burchfield, et al. 2020) suggests that lightning likely kills hundreds of millions of tropical trees annually, and could amount to 5%–10% of tropical forest biomass mortality (Gora et al. 2025b). Furthermore, lightning occurs within the context of convective storms (Williams 2005), and thus strong winds and windthrow events are expected to co‐occur with lightning. Using lightning as a proxy for storm activity, Gora et al. (2025b) found that lightning frequency was a strong predictor of pantropical spatial variation in carbon stocks, comparable in importance to maximum temperature and water availability.

Lightning also is likely to affect species composition and forest gap formation. Richards et al. (2022) found strong interspecific variation in lightning tolerance, with species most likely to be struck also having the highest probability of survival. Species‐level differences in lightning tolerance are particularly important because they suggest that forests can compositionally acclimate to differences in lightning frequency, thereby lessening the effects of higher lightning frequency on forest turnover rates. Furthermore, lightning strikes typically create forest gaps because their damage propagates through tree crowns across areas up to 2000 m^2^ (mean = 451 m^2^), damaging and killing both directly struck trees and their neighboring trees (lightning, on average, kills 5.3 trees, and damages an additional 18.3 trees per strike) (Yanoviak et al. 2017, 2020; Gora and Esquivel‐Muelbert 2021). Accordingly, lightning is a major gap forming agent that likely has corresponding effects promoting gap‐specialist abundance. These findings suggest that variation in lightning frequency over time will shape forest composition, structure, and function, but we lack the large‐scale, long‐term field data that would be required to empirically test these landscape‐level effects.

A species‐specific and spatially explicit model that simulates lightning can address these knowledge gaps. There is promise for such an approach as demonstrated in recent studies that have parameterized lightning mortality risk models from in situ observations at BCI (Gora, Muller‐Landau, et al. 2020; Richards et al. 2022). Incorporation of lightning into a more comprehensive forest dynamics model is now needed to improve the accuracy of tree mortality estimates, including the dependence of mortality on tree size, and tree traits. Furthermore, forest dynamics models can predict how community‐level dynamics will vary in response to changes in strike frequency.

Direct lightning frequency trend detection on regional scales is difficult because observational time series are of limited duration (~15 years; Thompson et al. 2014), the accumulated observation duration at any location is small (Cecil et al. 2015), and there is substantial interannual variability (Clark and Cecil 2024). However, various regional proxies indicate that lightning frequency has been increasing. From 1975 to 2017, the number of days during which thunder was recorded by meteorological stations more than doubled across the Amazon and increased by 20%–50% across Central America and the Congo Basin (Lavigne et al. 2019). These changes in thunder day occurrence are positively correlated with flash density over most of the tropics, though some regions (notably western Africa and northern India) are exceptions (Lavigne et al. 2019). Satellite measurements captured strong increases in the intensity and extent of convective storms across the Congo Basin from 1982 to 2016 (Raghavendra et al. 2018). Convective available potential energy (CAPE) has also increased across the Amazon from 1985 to 2020 (Urquiza‐Muñoz et al. 2024). Several climate modeling studies also suggest that lightning will increase in the future over tropical land (Price and Rind 1994; Banerjee et al. 2014). Other modeling studies found regional variability in the response of lightning frequency to increased temperature (Charn and Parishani 2021), and others have emphasized the possibility of regional decreases in lightning (Finney et al. 2018). Simulation of how forests will respond to changes in lightning frequency requires explicit representation of lightning in models.

Here, we incorporate lightning‐caused mortality into a forest dynamics model to answer three questions. First, how well does a lightning‐enabled model simulate field observations under current conditions? Second, how do changes in lightning frequency affect forest AGB? Third, do differences in tree tolerance to lightning allow forests to compositionally acclimate to changes in lightning disturbance regimes? Answering these questions will yield a framework for investigating lightning disturbance in silica in a manner that is impossible with existing in situ data.

## 2 Methods

### 2.1 Model Description

We used the TROLL model version 3.1 in this study (Chave 1999; Maréchaux and Chave 2017). TROLL is an individual‐based and spatially explicit model of forest dynamics. TROLL has been evaluated in a number of recent studies of tropical forests and has generally performed well against observed benchmarks (Chave 1999; Maréchaux and Chave 2017; Fischer et al. 2019; Schmitt et al. 2020; Rau et al. 2022; Schmitt et al. 2025). The model discretizes space into 1 × 1 × 1 m voxels. We simulated a spatial area of 200 × 200 m. The height of the simulated domain was 50 m, which was large enough to accommodate the tallest trees at our site. Each 1 m^3^ ground‐level voxel could be associated with at most 1 tree. Thus, the maximum number of trees in our simulation was 40,000. All trees were characterized by their species assignment, diameter at breast height (DBH), and leaf area. Species‐specific allometric equations related DBH to tree height and crown radius (Martínez Cano et al. 2019) and tree height to crown depth (Maréchaux and Chave 2017). Tree crowns spread over three‐dimensional space and assume an umbrella shape (Schmitt et al. 2023). Lianas were not included in this version of TROLL.

Growth, recruitment, and mortality were the main processes simulated by the model. Growth was driven by the balance of photosynthesis and respiration. Before computing photosynthesis, the model computed the photosynthetic photon flux density absorbed by each crown using the Beer–Lambert law (Maréchaux and Chave 2017) and stomatal conductance was calculated using the optimization approach of Medlyn et al. (2011). Then, gross photosynthesis was calculated as the minimum of a rate limited by Rubisco activity or a rate limited by RuBP regeneration (Farquhar et al. 1980). Leaf maintenance respiration per unit leaf area was treated as a function of temperature and species‐specific traits (Atkin et al. 2015). Stem maintenance respiration was assumed to be proportional to sapwood volume (Ryan et al. 1994). Belowground respiration was scaled to aboveground respiration (Maréchaux and Chave 2017). Growth respiration was set to be proportional to the difference between gross photosynthesis and maintenance respiration (Thornley and Cannell 2000). Tree‐level net primary production (NPP) was computed as the difference between gross photosynthesis, maintenance respiration, and growth respiration. Below a species‐specific size threshold, fixed proportions of NPP were allocated to wood and canopy biomass. Above that size threshold, the amount of NPP allocated to wood was reduced, reflecting empirical evidence for a size‐related relative growth decline in large trees (Stephenson et al. 2014; Maréchaux and Chave 2017). After the model updated wood biomass, it used allometric equations to update stem volume, DBH, height, crown radius, and crown depth.

Recruitment was modeled as a stochastic process. A seedling bank was present in all ground‐level 1 m^3^ voxels where trees were not already present. Each seedling bank was filled by external seed rain and by seeds generated by mature trees from within the simulated domain. Then, on each monthly timestep, the model identified all the unoccupied surface‐level voxels receiving enough light for a tree to have positive net primary production on an average day. For each of these voxels, a species from the corresponding seedling bank was randomly selected to recruit and was assigned a DBH of 1 cm.

Before including lightning, tree‐level mortality was treated as a two‐stage stochastic process. In the first stage, the model calculated the probabilities of mortality due to background mortality and carbon starvation. Background mortality probability was a species‐specific constant based on wood specific gravity (Kraft et al. 2010; Wright et al. 2010). The probability of carbon starvation was set to one if the consecutive months with NPP < 0 exceeded the species' leaf lifespan and was zero otherwise. The sum of background and carbon starvation mortality probabilities then constituted the first‐stage mortality probability. We then sampled from a uniform probability density function, bounded between 0 and 1, and simulated mortality if the sample was less than the probability of mortality. The second stage of mortality was driven by treefall. For each tree, the probability of treefall was set to be an increasing function of tree height. Again, mortality was simulated if a random sample from a uniform probability density function (bounded between 0 and 1) was less than the probability of mortality (Maréchaux and Chave 2017). If treefall was indicated, further random numbers were drawn to determine the direction of treefall, and to determine whether any trees along the direction of primary treefall experienced secondary treefall (Van Der Meer and Bongers 1996).

### 2.2 Implementation of Lightning

We implemented lightning by adding a third stage to the mortality process. First, we defined a cloud‐to‐ground lightning flash (CG fl) frequency per unit area (*λ*). At BCI, *λ* is 12.7 CG fl km^−2^ years^−1^ (95% confidence interval: 10.9–14.5 CG fl km^−2^ years^−1^) (Yanoviak et al. 2020). On every monthly timestep, every 1 m^3^ surface voxel was assigned a strike probability consistent with *λ*, and a random number was drawn to determine if a strike occurred. If a strike occurred, we searched the local 15 m radius region for the tallest tree, and targeted that tree for the strike. This approach to modeling direct‐strike likelihood is consistent with empirical observations of lightning strikes to what is typically the tallest tree in a local area (Gora, Muller‐Landau, et al. 2020).

Empirical models for lightning mortality risk already exist (Gora, Muller‐Landau, et al. 2020; Richards et al. 2022). However, with 4–6 empirical parameters, they would add significantly to the computational costs of model uncertainty and sensitivity analyses. Such complexity may not be justified because dynamical models typically have very few consequential parameters (Saltelli et al. 2000; Pappas et al. 2013; Puy, Beneventano, et al. 2022; Schmitt et al. 2023). Thus, we developed a new community‐level representation of lightning risk with fewer parameters. We assumed that the probability of mortality increases with tree size and decreases with distance from the strike relative to a “direct‐hit” baseline (Yanoviak et al. 2020; Richards et al. 2022). These properties can be satisfied with a 2‐parameter equation:

(1) $$\log_{10}\frac{P_{\mathrm{com}}\left(r,D\right)}{P_{\text{direct}}}=-a\left(\frac{70\;\mathrm{cm}}{D}\right)\left(\frac{r}{45\;m}\right)$$

Here, *P*_com_(*r*, *D*) is the community‐level probability of mortality of a tree of DBH *D* (units: cm) at a distance *r* (units: m) from the strike. The two model parameters are *P*_direct_ (mortality probability of a direct strike; dimensionless) and the exponential decay rate *a* (dimensionless). For simplicity, our new model does not account for sub‐lethal crown damage and all lightning‐killed trees die instantaneously and the space becomes available for recruitment. In the remainder of the paper, we will refer to Equation 1 as a “Community Level Lightning Risk (CLLR) model.”

To parameterize Equation 1, we binned the lightning mortality data of Yanoviak et al. (2020) into 5 distance classes and 3 DBH classes. These data required careful interpretation because lightning was observed to cause significant crown damage in a large number of trees (Yanoviak et al. 2020), and we do not know the proportion of trees for which this damage was ultimately lethal because mortality rates remained elevated beyond the timeline of this study (Gora et al. 2025a). We therefore introduced a parameter, *d* (ranging from 0 to 1), which specified the proportion of lightning‐damaged trees that eventually died due to the strike. This parameter was important for model calibration because it determined the observed number of dead trees that the calibrated model would simulate. The same value of *d* was assumed to apply to all DBH and distance classes. With *d* defined, we calculated the scaled residuals using the observations from Yanoviak et al. (2020):

(2) $$R\left(a,P_{\text{direct}},d\right)=\sum_{r=1}^{5}\sum_{D=1}^{3}\frac{{\left(M_{D,r}\left(a,P_{\text{direct}}\right)-O_{D,r}\left(d\right)\right)}^{2}}{O_{D,r}\left(d\right)}$$

*M*_*D,r*_ represents the predicted number of mortalities in each DBH and distance class using the probabilities from Equation 1 and the numbers of trees from Yanoviak et al. (2020). *O*_*D,r*_ represents the number of observed mortalities in each DBH and distance class. We took *d* to be a free parameter and then calibrated the parameters *a* and *P*_direct_ by minimizing Equation 2. Minimization was done by carrying out an exhaustive search with resolution in *a* of 0.06 (−7 ≤ *a* ≤ −1) and a resolution in *P*_direct_ of 0.01 (0 ≤ *P*_direct_ ≤ 1). We then evaluated the goodness‐of‐fit by visual inspection and computation of the bias and RMS error (Supporting Information S1).

We then developed a generalization of Equation 1 to account for interspecific differences in lightning tolerance (Richards et al. 2022). For a tree of species *i*, the probability of lightning mortality (*P*_*i*_) was set according to:

(3) $$\log_{10}\frac{P_{i}}{1-P_{i}}=\log_{10}\frac{P_{\mathrm{com}}}{1-P_{\mathrm{com}}}+{∆}_{i}$$

Thus, this generalization corresponds to a shift in the log‐odds with respect to the community‐level model (Equation 1). The log‐odds shifts, ${∆}_{i}$, were taken from the empirical results of Richards et al. (2022) and are listed in Supporting Information S2. A positive value of ${∆}_{i}$ results in *P*_*i*_>*P*_com_ and a negative value of ${∆}_{i}$ results in *P*_*i*_<*P*_com_. Working in log‐odds space has the advantage of keeping all probabilities between 0 and 1; however, ${∆}_{i}$ can be any real number. A species that survived every time it was struck would have ${∆}_{i}$ approaching negative infinity, and a species that died every time it was struck would have ${∆}_{i}$ approaching positive infinity. Values of ${∆}_{i}$ were available for only about 25% of all species parameterized in the model. For species without measured ${∆}_{i}$, we set ${∆}_{i}=0$. In the remainder of the paper, we will refer to Equation 3 as a “Species‐Specific Lightning Risk (SSLR) model.”

To summarize, the steps involved in parameterization included: (i) sample a value for *d*, between 0 and 1, in order to quantify the target observations; (ii) define our CLLR model by finding the values of *a* and *P*_direct_ that minimize Equation 2; (iii) defining our SSLR model by using (Equation 3), the CLLR model, and the observed ${∆}_{i}$.

### 2.3 Model Inputs

The required model inputs included meteorological variables, species definitions, and global parameters. The meteorological variables included half‐hourly photosynthetic photon flux density, temperature, and vapor pressure deficit. We obtained these variables from a 16‐year (1997–2012) meteorological dataset based on in situ climate observations at Barro Colorado Island available through the Smithsonian Tropical Research Institutes' Physical Monitoring Program and processed according to Levy‐Varon et al. (2019). For each half hour of each month, we computed the 16‐year average. The model was then forced with these average values for each simulated year.

The model contained approximately 20 additional parameters (not species‐specific) that were specified in an input file (Medvigy et al. 2025). Some of these parameters were used to complete the parameterizations for leaf physiology, allocation, mortality, and seed rain. Others provided further simulation configuration details (how long to run the simulation, timestep, etc.). Full lists of model equations and parameter definitions are provided in Maréchaux and Chave (2017).

The model defined species according to the traits listed in Supporting Information S2. In order to fully define a species, the model required leaf mass per unit area (LMA), wood specific gravity, leaf %N, leaf %P, three allometric parameters that defined the DBH‐H relationship, and a DBH threshold that determined reproductive maturity. All of the allometric parameters and wood specific gravity were taken from site level observations at BCI (Martínez Cano et al. 2019). Values for LMA, leaf %N, and leaf %P were species‐level averages derived from observations reported in the TRY database (Kattge et al. 2020). These values were not necessarily tied to BCI. Collectively, these datasets enabled complete parameterizations for 96 species. These 96 species included 20 of the 27 species with measured lightning tolerances (${∆}_{i}$), and 9 of the 11 species with a lightning tolerance that was significantly different from the community mean (Richards et al. 2022).

We augmented this species list with the remaining 7 species with measured lightning tolerances (Table S1). For these 7 species, the only trait that we lacked was leaf %P. The model used leaf %P only in the calculation of three further parameters: the maximum rate of carboxylation, the maximum rate of electron transport, and the rate of leaf dark respiration. For the seven species only, we used alternative calculations for these physiological parameters (Supporting Information S2).

### 2.4 Parameter Uncertainty Analysis

We propagated parameter uncertainty to model output uncertainty (Table 1). Previous work identified the parameters to which the no‐lightning TROLL was most sensitive (Schmitt et al. 2023). These parameters included the apparent quantum yield for carbon fixation (*ϕ*), maximum basal mortality rate (*m*_0_), fraction of NPP allocated to wood growth (*f*_wood_), fraction of NPP allocated to canopy (*f*_canopy_), and allometric parameters related to crown radius. One complication is that *f*_wood_ and *f*_canopy_ satisfy a constraint, *f*_wood_ + *f*_canopy_ + *f*_below_ = 1, where *f*_below_ represents belowground allocation. Thus, *f*_wood_ and *f*_canopy_ are not independent. We addressed this complication by transforming the problem into cylindrical coordinates, so that *f*_below_ is defined along the axial direction and another parameter, *θ*, is the angle between the *f*_wood_ and *f*_canopy_ axes ($\theta =\tan^{-1}\frac{f_{\text{canopy}}}{f_{\text{wood}}}$). We chose not to include the crown radius allometric parameters in our uncertainty analysis because, unlike Schmitt et al. (2023), we had access to a local crown area allometric parameterization and thus its associated uncertainty was expected to be relatively small (Martínez Cano et al. 2019). Thus, we included analysis of *ϕ*, *m*_0_, *f*_below_, and *θ* for all versions of TROLL. For simulations with lightning, we also included *λ* (lightning frequency) and *d* (fraction of trees that were observed to be damaged by lightning and assumed to eventually die due to the strike). We then repeatedly sampled the distributions of input parameters and ran the model for each sample. Finally, we calculated the statistics of the distributions of model outputs. We determined the effects of lightning by comparing results from a no‐lightning TROLL model, CLLR‐enabled TROLL, and SSLR‐enabled TROLL. Details of our uncertainty analysis are given in Supporting Information S3.

**TABLE 1:** Model parameters considered in the uncertainty analysis.

Parameter	Description	Units	Range of values	Used in no‐lightning TROLL	Used in CLLR and SSLR TROLL
*ϕ*	Apparent quantum yield for carbon fixation	mol C (mol photon)^−1^	(0.04, 0.09)	Yes	Yes
*m*_0_	Maximal baseline mortality rate	Years^−1^	(0, 0.03)	Yes	Yes
*f*_below_	Fraction of NPP allocated belowground	Dimensionless	(0.1, 0.5)	Yes	Yes
*θ*	Azimuthal angle with *f*_wood_ on *x*‐axis and *f*_canopy_ on *y*‐axis	Radians	$\left(\frac{\pi }{12},\frac{5\pi }{12}\right)$	Yes	Yes
*λ*	Frequency of cloud‐to‐ground lightning flashes	CG fl km^−2^ years^−1^	(10.9, 14.5)	No	Yes
*d*	Proportion lightning‐damaged trees that die as a result of the strike	Dimensionless	(0, 1)	No	Yes

**FIGURE 1:**
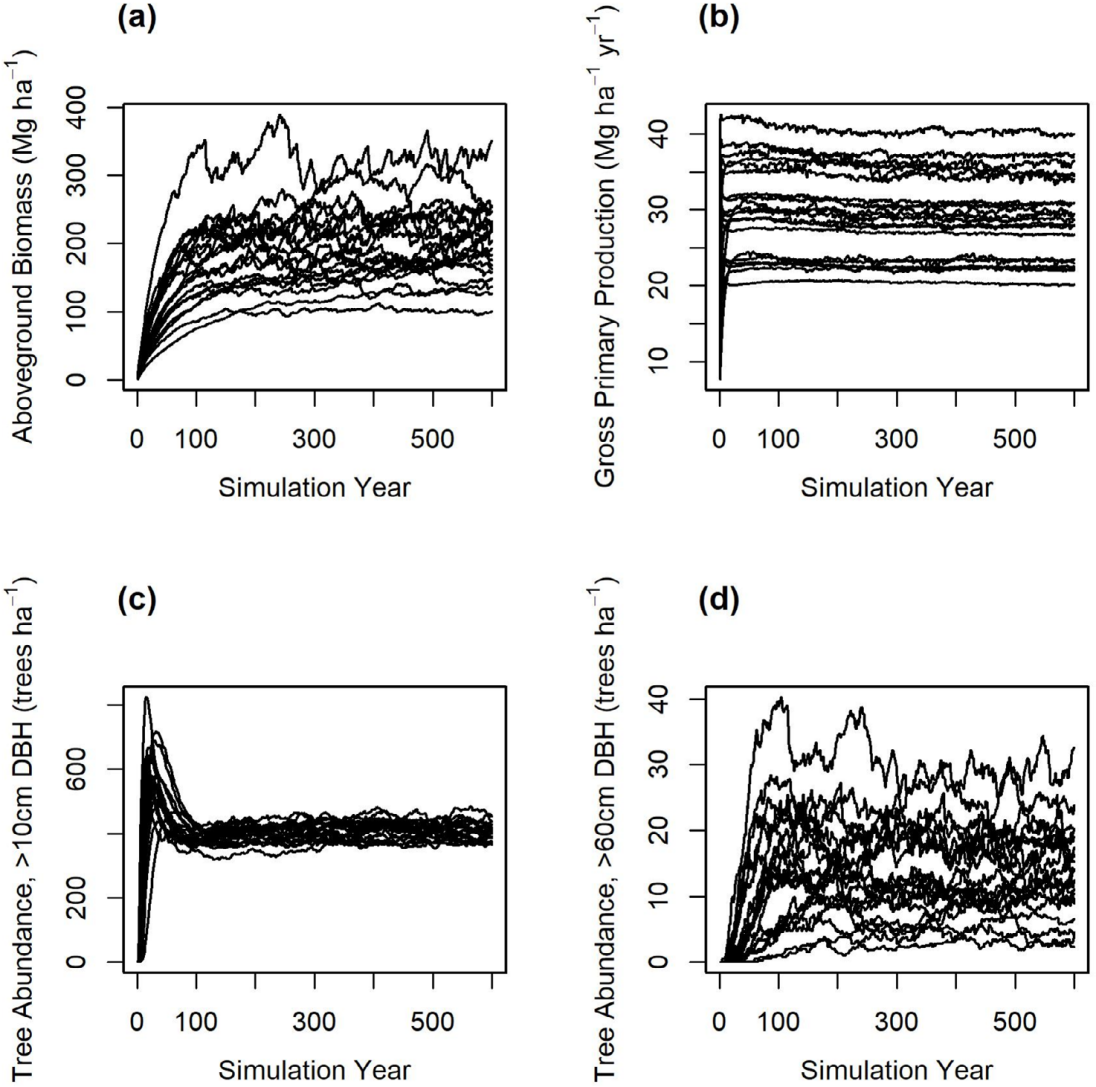
Model outputs from 20 TROLL simulations. The 20 simulations had different parameter values which corresponded to the first 20 elements of the Sobol’ sequence. The results show an approach to dynamic equilibrium for (a) AGB, (b) GPP, (c) N10, and (d) N60.

In contrast to Schmitt et al. (2023), we carried out our analysis entirely with the native TROLL model and not an emulator. One 600‐year simulation was done for each parameter set. The duration was sufficient to allow domain‐averaged state variables to reach their steady‐state values (Figure 1). For each simulation, model outputs included community‐ and species‐level aboveground biomass (AGB), gross primary productivity (GPP), and the numbers of trees with DBH > 10, > 30, and > 60 cm (N10, N30, and N60, respectively). We also recorded the number of lightning strikes, lightning mortalities, and lightning survivorship by species and size class. All outputs were averaged over the last 50 years of each simulation.

### 2.5 Model Evaluation

We compared our model results to observations. Observed AGB was based on census data from BCI (Hubbell et al. 1999) and taken from Meakem et al. (2018). AGB uncertainty, GPP, and GPP uncertainty were taken from Koven et al. (2020). Values for N10, N30, and N60 were derived from the 2005 census data of Condit et al. (2019). 95% confidence intervals were obtained by bootstrapping the census data.

### 2.6 Changes in Lightning Frequency

There is substantial uncertainty in how tropical forest lightning strike frequency may change in the coming decades (Price and Rind 1994; Banerjee et al. 2014; Finney et al. 2018; Charn and Parishani 2021). We therefore carried out an additional uncertainty analysis over a plausible range of values to better understand how future changes in lightning could affect forest dynamics at BCI. Our procedure was to first select 15 strike frequencies: 9, 10, 11, 12, 13, 14, 15, 16, 17, 18, 19, 20, 21, 22, and 23 CG fl km^−2^ years^−1^. Given that this range is far broader than the current‐day 95% confidence range on strike frequency (Table 1), additional simulations were required. We constructed an ensemble of simulations for each strike frequency defined by the other five parameters of our previous uncertainty analysis (*ϕ*, *m*_0_, *f*_below_, *θ*, *d* in Table 1). We found that the means and variances of the distributions of model outputs converged to within 5% after fewer than 1000 sampled parameter sets; thus, we sampled the parameter distributions 1000 times. This procedure led to 15,000 (15 strike frequencies × 1000 parameter sets) simulations. The same parameter combinations were used for each strike frequency.

A limitation of this approach is that we did not expect all parameter sets to be realistic with respect to current‐day observations. We therefore generated another simulation ensemble to determine whether any manifestly poor choices for parameters might have biased our results. To this end, we randomly chose 1000 of the parameter sets that led to realistic current‐day AGB, GPP, N10, and N60 (i.e., within their respective 95% confidence intervals) when the model was forced with the current‐day *λ*. For each of these 1000 parameter sets, we ran the model with the same 15 strike frequencies from 9 to 23 CG fl km^−2^ years^−1^. Thus, this ensemble only includes members that performed reasonably well under current climate.

Our main focus is on forests at equilibrium (e.g., after 600 years of simulation). However, we also briefly considered the transient dynamics following a change in lightning frequency. Our approach is described in Supporting Information S4.

## 3 Results

### 3.1 Impact of Lightning Under Current Climate Conditions

**FIGURE 2:**
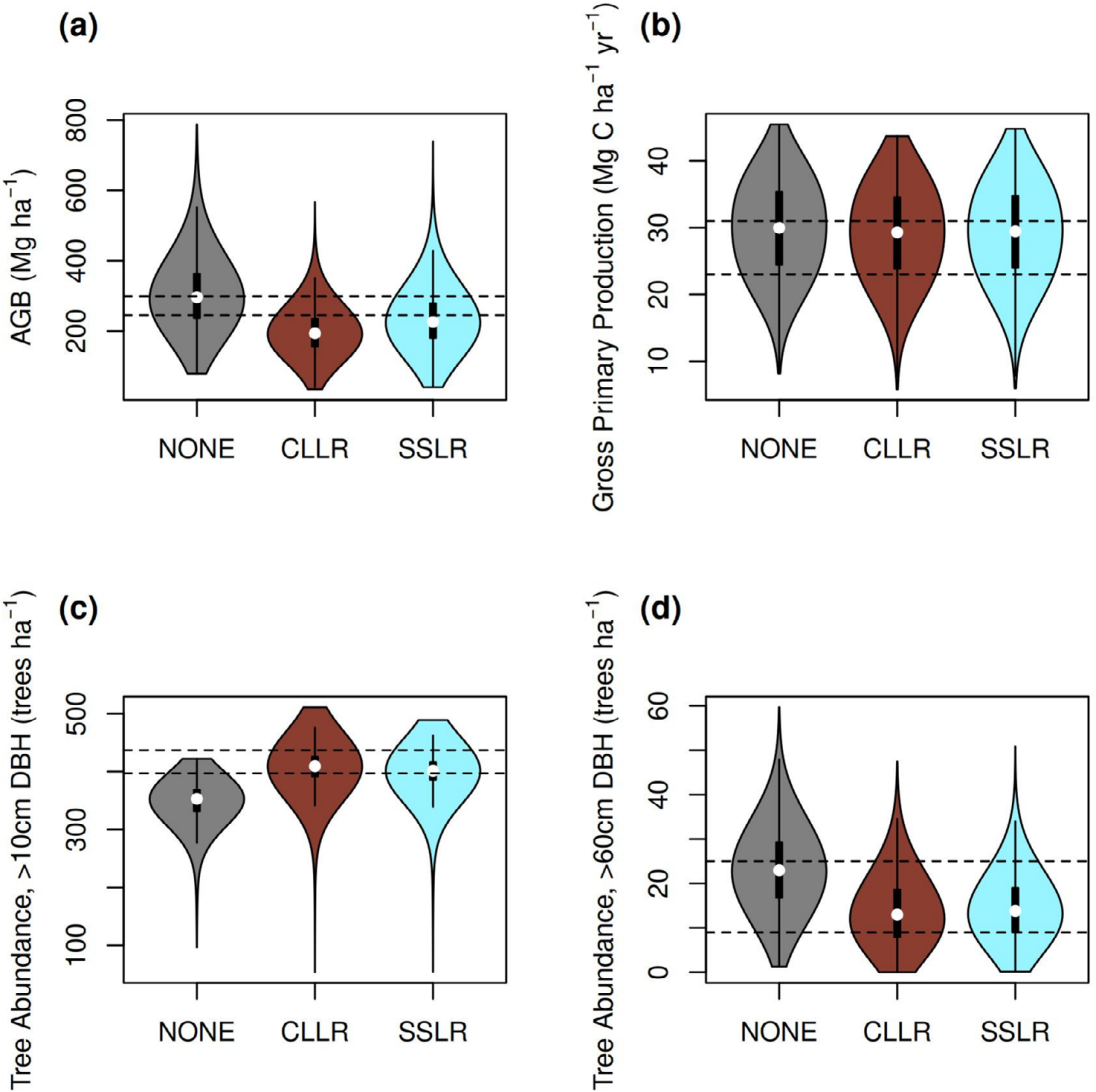
TROLL model evaluation for different implementations of lightning. The dashed black lines indicate the 95% confidence interval on the observations. The “NONE” distributions correspond the no‐lightning model, the “CCLR” distributions correspond to TROLL with a community‐level lightning risk model, and the “SSLR” distributions correspond to TROLL with a species‐specific lightning risk model. (a) Aboveground biomass (AGB). (b) Gross primary production (GPP). (c) Number of trees with DBH > 10 cm (N10). (d) Number of trees with DBH > 60 cm (N60).

We compared simulated and observed values of AGB, GPP, number of trees with DBH > 10 cm (N10), and number of trees with DBH > 60 cm (N60) (Figure 2). This comparison was made in the context of three TROLL model versions: no incorporation of lightning, incorporation of the community‐level lightning risk (CLLR) model (Equation 1), and incorporation of the species‐specific lightning risk (SSLR) model (Equation 3). AGB is shown in Figure 2a. All simulated distributions of AGB showed strong support for the observed AGB. However, the simulated distributions also had much larger uncertainties than the observations, and thus many simulated values fell outside the range of the observational uncertainty. Comparing the TROLL model versions amongst themselves, the no‐lightning simulations had the largest AGB, the CLLR TROLL had the smallest, and the SSLR TROLL was intermediate. All simulated distributions of GPP also showed strong support for the observations (Figure 2b), and lightning had little effect on simulated GPP. N10 tended to be underestimated by the no‐lightning simulations, but was better simulated by both the CLLR and SSLR TROLL (Figure 2c). Observed and simulated distributions of N60 had substantial overlap (Figure 2d), but the no‐lightning simulations tended to have larger N60 than either CLLR TROLL or SSLR TROLL. We also evaluated distributions of N30 (Supporting Information S5). Observations fell within the simulated range and there was little difference across model formulations.

**FIGURE 3:**
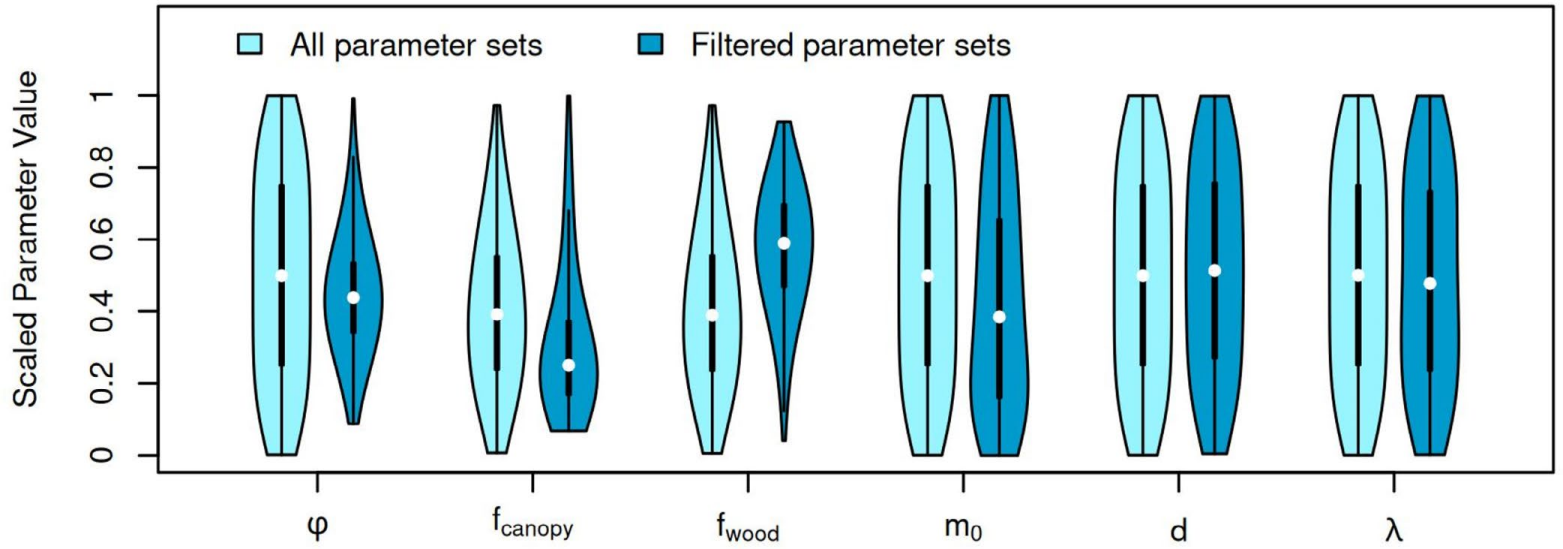
Parameter distributions for all parameter sets, as well as for only those parameter sets that were filtered to be consistent with observed AGB, GPP, N10, and N60. All parameter values were linearly scaled to facilitate comparison. A scaled value of 0 represents the minimum parameter value and a scaled value of 1 represents the maximum parameter value (the maximum and minimum values are given in Table 1). In particular, note that the range of λ corresponds only to the current‐day uncertainty interval. Results are shown for the species‐specific lightning risk (SSLR) version of TROLL.

We then identified those parameter combinations for which the simulations were consistent with the observations. Specifically, we required that the simulated values of AGB, GPP, N10, and N60 all be within the observational uncertainty. Thus, different parameter sets were selected for the no‐lightning, CLLR, and SSLR formulations of TROLL (Supporting Information S6). Univariate projections of our results are shown in Figure 3 for the SSLR TROLL. The selected parameter sets generally had smaller *ϕ*, smaller *f*_canopy_, larger *f*_wood_, and smaller *m*_0_ than our original, unfiltered parameter sets. The distributions of *d* and λ were very similar.

### 3.2 Forest Responses to Changes in Lightning Strike Frequency

**FIGURE 4:**
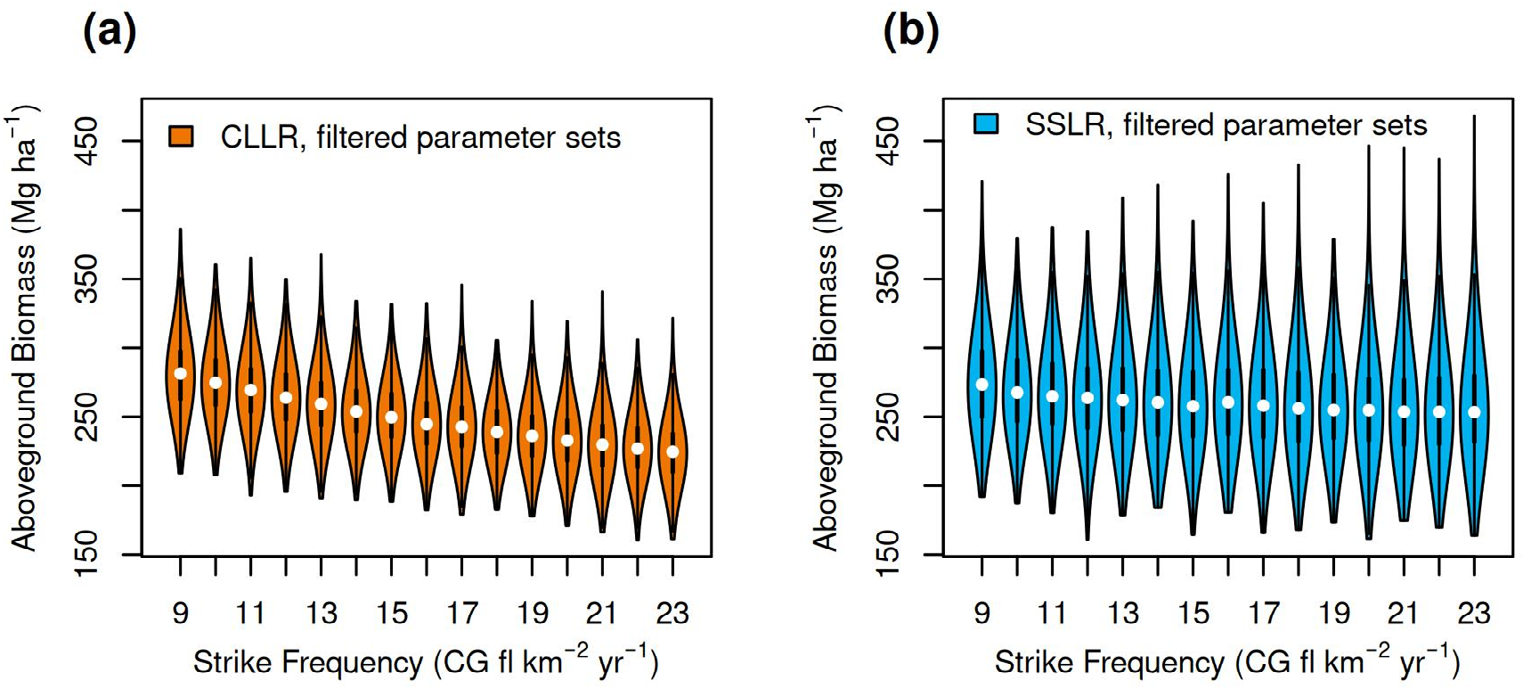
Responses of aboveground biomass to potential future changes in lightning strike frequency. The “CLLR” distribution (panel a) corresponds to the community‐level lightning risk TROLL and the “SSLR” distribution (panel b) corresponds to the species‐specific lightning risk TROLL. In both panels, the parameter sets were limited to those that led to consistency with current‐day observations.

We then used the model to evaluate how AGB would respond to plausible future lightning frequencies (*λ*). Overall, AGB declined with increasing *λ* (Figure 4). However, the decline was much steeper in CCLR TROLL than in SSLR TROLL. Further, the response appeared nonlinear, especially for the SSLR simulations. Thus, a reduction in lightning frequency had more of an effect than an increase in lightning frequency.

To understand why *λ* had a stronger negative effect on AGB for CLLR TROLL than for SSLR TROLL, we calculated species‐ and *λ*‐specific AGB, averaged over all (filtered) parameter sets. We defined a “lightning scaling factor” for each species, *s*_*i*_, according to:

$$s_{i}=\frac{1}{1+e^{-{∆}_{i}}}$$

*s*_*i*_ is a convenient because it is bounded between 0 and 1, and can be interpreted as the mortality risk of the SSLR model when the mortality risk of the CCLR model is 0.5. Thus, a value of *s*_*i*_ < 0.5 would indicate relative lightning tolerance and a value of *s*_*i*_ > 0.5 would indicate relative lightning intolerance (*s*_*i*_ = 0.5 marks neutrality, and also is the default value for those species with unknown tolerance).

**FIGURE 5:**
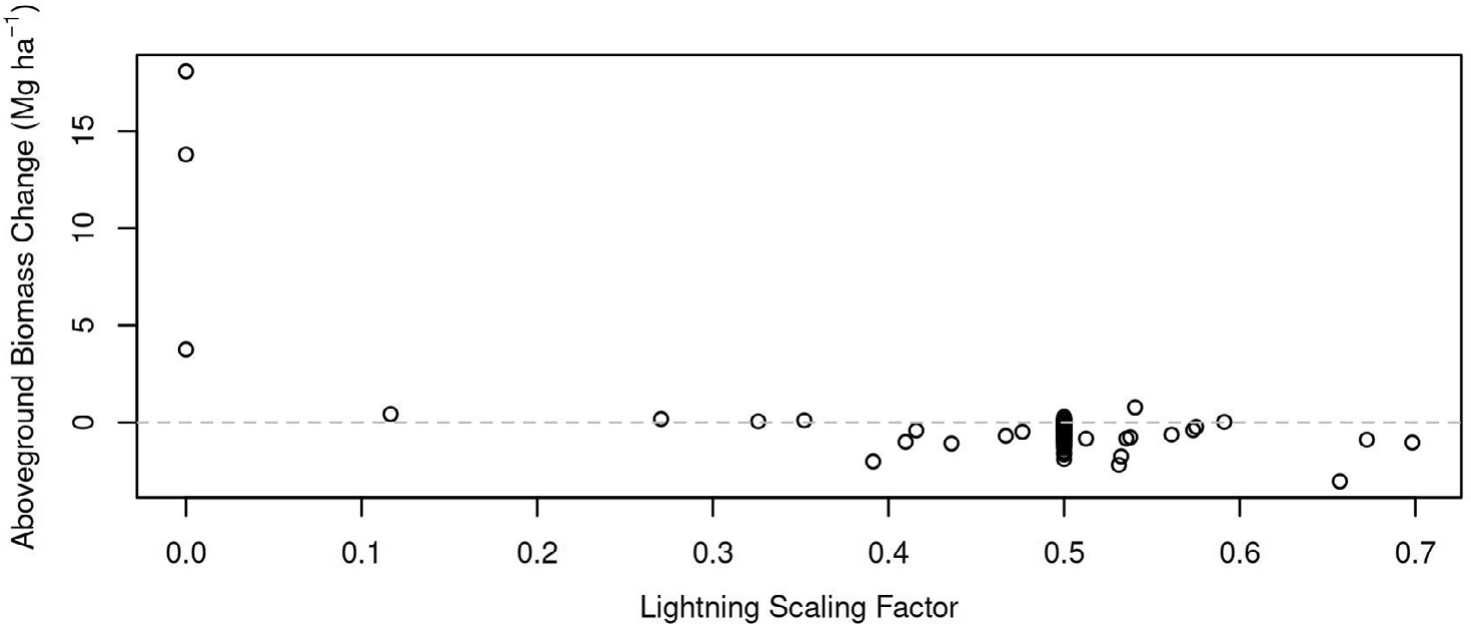
Effect of lightning frequency (*λ*) on species‐level aboveground biomass. Lightning scaling factors (*s*_*i*_) indicate species that are tolerant (*s*_*i*_ < 0.5), neutral (*s*_*i*_ = 0.5), or intolerant (*s*_*i*_ > 0.5). The species‐level aboveground biomass change corresponds to the difference AGB (*λ* = 23 CG fl km^−2^ years^−1^) minus AGB (*λ* = 9 CG fl km^−2^ years^−1^).

**FIGURE 6:**
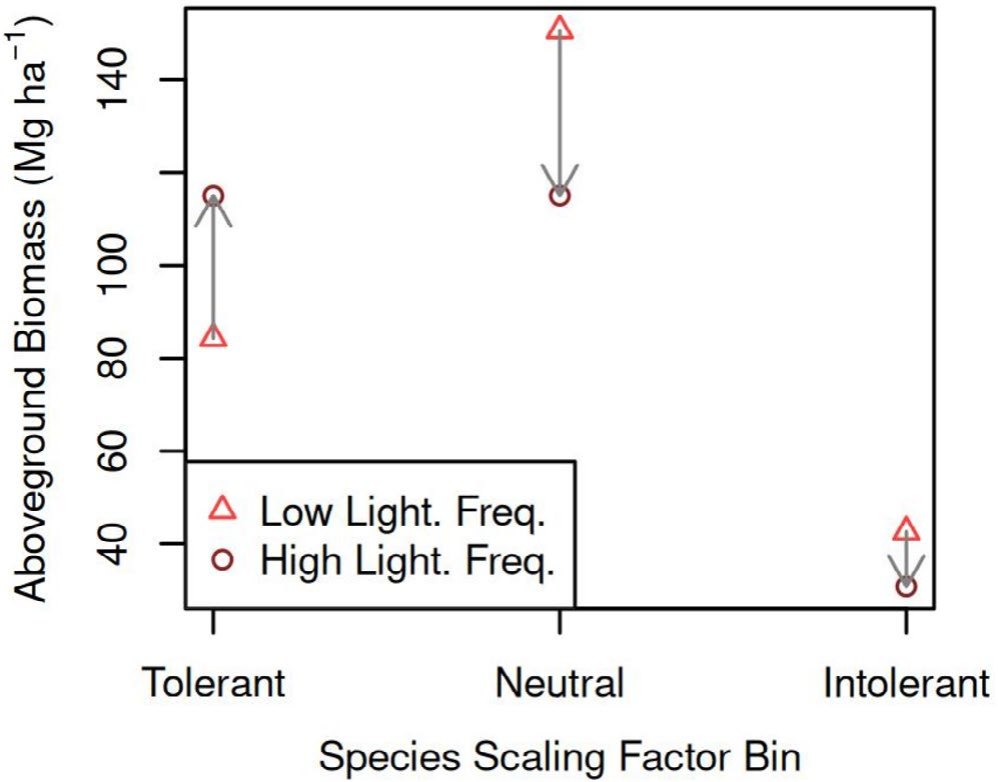
Effect of lightning frequency (*λ*) on aboveground biomass of lightning‐tolerant, neutral, and intolerant species. “Low Light. Freq.” corresponds to *λ* = 9 CG fl km^−2^ years^−1^ and “High Light. Freq.” corresponds to *λ* = 23 CG fl km^−2^ years^−1^. Species were binned according to their lightning scaling factors (*s*_*i*_): Tolerant (*s*_*i*_ < 0.5), neutral (*s*_*i*_ = 0.5), or intolerant (*s*_*i*_ > 0.5).

Comparing species‐level AGB for *λ* equal to 9 and 23 CG fl km^−2^ years^−1^, we see that the AGB of lightning‐tolerant species is generally larger at larger *λ*, and that the AGB of lightning‐intolerant species is generally smaller (Figure 5). Neutral species have mixed results. What particularly stands out is the strong sensitivity of two lightning‐tolerant species: the AGB of *Dipteryx oleifera* increased by 18 Mg ha^−1^ (the largest increase) and the AGB of *Hura crepitans* increased by 14 Mg ha^−1^ (the second‐largest increase). When species are binned together as either tolerant, neutral, or intolerant, we found that the AGB increases of the tolerant species nearly balanced the AGB decreases of neutral and intolerant species (Figure 6).

**FIGURE 7:**
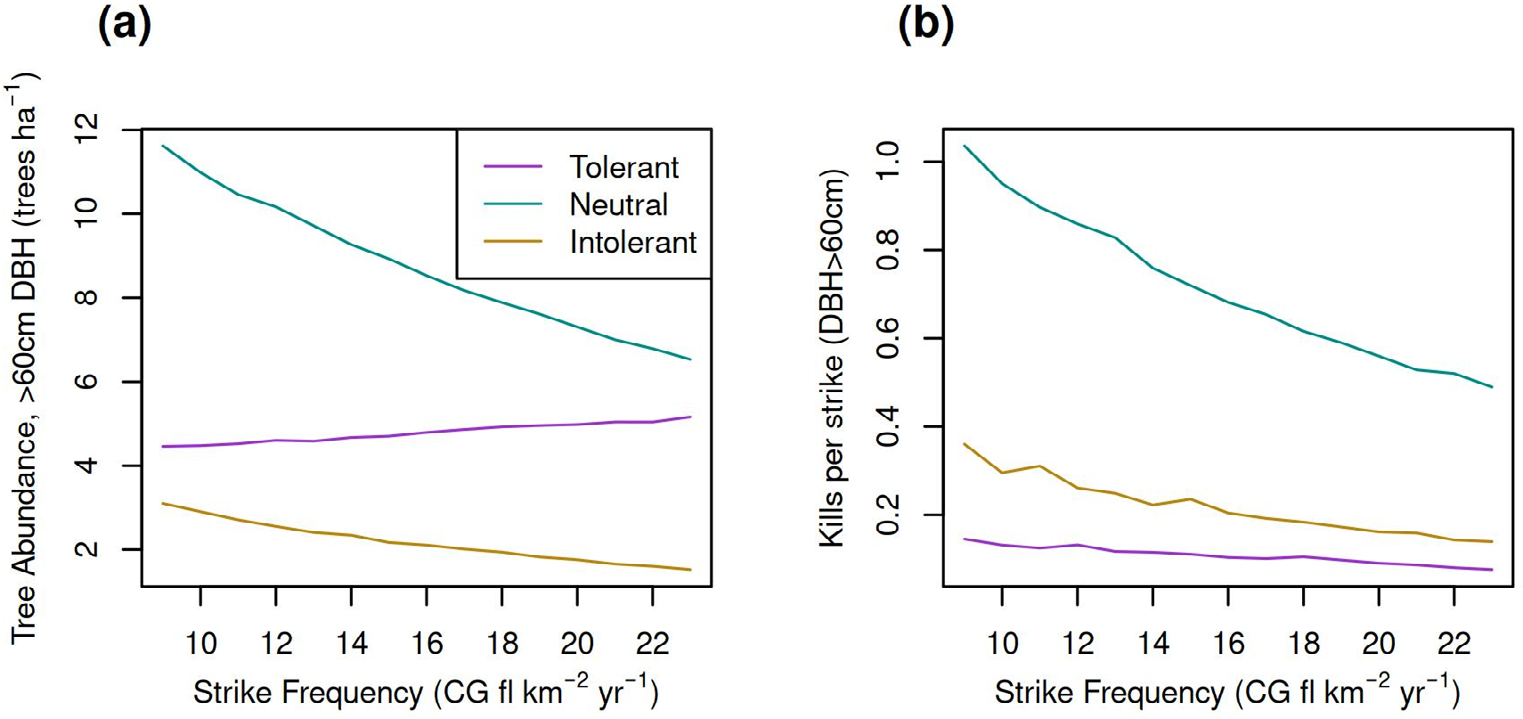
Variation of (a) N60 and (b) number of trees (DBH > 60 cm) killed per strike with *λ*. Species were binned according to their lightning scaling factors (*s*_*i*_): Tolerant (*s*_*i*_ < 0.5), neutral (*s*_*i*_ = 0.5), or intolerant (*s*_*i*_ > 0.5).

We next investigated the apparent non‐linearity in the median response of AGB to *λ* (Figure 4). We expected that increasing *λ* would disproportionately affect the number of neutral and intolerant trees. Thus, we computed N60, conditional on *λ* and species. We again binned the species by *s*_*i*_ (tolerant, neutral, intolerant) and averaged the results from the filtered parameter sets. Figure 7a shows that the N60 for neutral and intolerant species decreases sharply as *λ* increases. Thus, as *λ* increases, the number of kills per strike declines because there are fewer killable trees (Figure 7b). The number of kills per strike also decreases slightly for the lightning‐tolerant trees, indicating that the number of modestly tolerant trees has also declined in favor of strongly tolerant species.

We also considered out‐of‐equilibrium conditions in which *λ* varied linearly in time (Supporting Information S4). We find that forest composition gradually adjusts to changes in lightning frequency, with increases in *λ* leading to larger AGB proportions of lightning‐tolerant species.

### 3.3 Effects of Parameter Uncertainty

**FIGURE 8:**
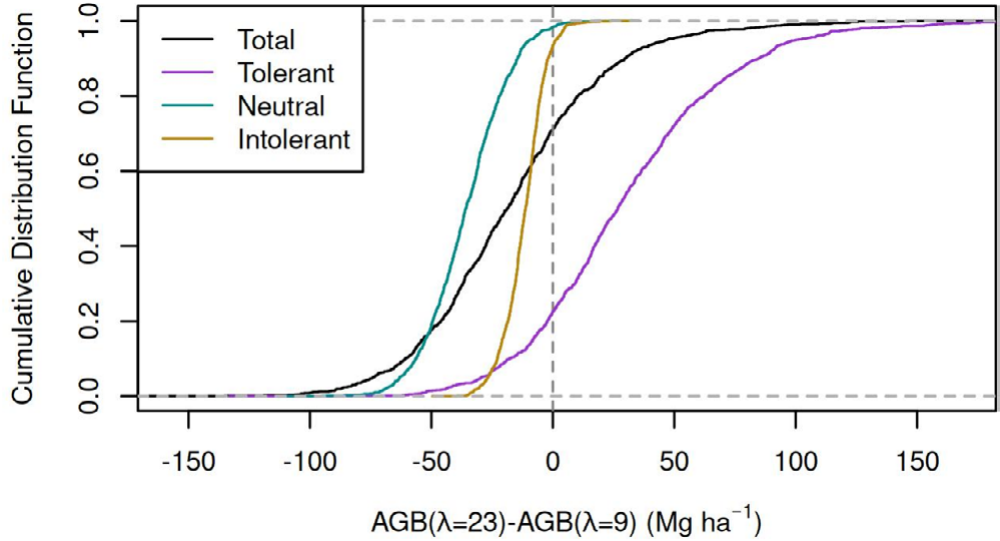
Effect of parameter uncertainty on the response of AGB to *λ*. The *x*‐axis shows the difference in AGB between simulations with *λ* = 23 CG fl km^−2^ years^−1^ and *λ* = 9 CG fl km^−2^ years^−1^. Species were binned according to their lightning scaling factors (*s*_*i*_): Tolerant (*s*_*i*_ < 0.5), neutral (*s*_*i*_ = 0.5), or intolerant (*s*_*i*_ > 0.5). Total AGB for all trees is also shown. The spread of the cumulative distribution function along the x‐axis reflects parameter uncertainty.

The results in Figures 5 and 6 correspond to averages over the filtered parameter sets. However, the choice of parameter set can strongly influence the results. Figure 8 shows the cumulative probability density function for the AGB difference (AGB_*λ*=23_–AGB_*λ*=9_) for the tolerant, neutral, and intolerant species. For more than 93% of the parameter sets, increasing *λ* causes the AGB of neutral and intolerant species to decline. But there is much more uncertainty in the case of tolerant species. On the one hand, increasing *λ* can cause tolerant species' AGB to increase so much that total AGB increases (29% of the parameter sets). However, the AGB of tolerant species (let alone total AGB) is not guaranteed to increase. Indeed, for 22% of parameter sets, increasing *λ* causes the AGB of tolerant species to decline.

## 4 Discussion

We have explicitly included lightning in a species‐specific, spatially explicit forest dynamics model. Model simulations allowed us to investigate plausible future scenarios without decades of monitoring or unrealistically large manipulative experiments. Thus, our model simulations provide a unique viewpoint of the mechanisms whereby lightning affects forest AGB, composition, and structure.

Our work is complementary to a recent study that used the LPJ‐GUESS model to simulate the effects of lightning on biomass and biomass mortality (Krause et al. 2025). That study also used the BCI lightning mortality dataset for model calibration and evaluation. However, these models are distinct in their scales of analysis. The TROLL model, used here, represents individual trees on a spatially explicit grid, and is species‐specific. Thus, TROLL can explicitly represent the effects of lightning flashover without additional assumptions regarding the spatial distribution of trees. Critically for this analysis, it can also represent species‐level differences in lightning tolerance. By contrast, LPJ‐GUESS represents age cohorts within patches but without explicit coordinates. It also adopts the plant functional type concept. This more aggregated structure supports model parsimony and computational efficiency, and makes LPJ‐GUESS well‐suited to global simulations and thus global assessments of lightning mortality (Krause et al. 2025).

### 4.1 Effects of Lightning on Forest Properties

Use of a model allows us to isolate the effects of lightning on forest processes without any confounding factors. Previous empirical work has shown that stem mortality rates exert a strong control on tropical forest AGB (Johnson et al. 2016; Suarez et al. 2023), and that even small changes in rates of stand‐replacing disturbances can have a large impact on AGB (Pugh et al. 2019). Because lightning is a mortality agent, we expected that the incorporation of lightning into a model would lead to reductions in simulated AGB. We also expected lightning to decrease the number of large trees given the vulnerability of large trees to lightning (Yanoviak et al. 2020; Gora, Muller‐Landau, et al. 2020). Our results were consistent with these expectations. However, we also found that GPP was scarcely affected by lightning, probably reflecting the limited effects of disturbance on productivity (Fu et al. 2017; Kunert et al. 2019).

Although increased lightning led to decreased AGB, the effect was relatively small. This may seem puzzling at first considering that lightning frequency is an important predictor of carbon stocks across forest inventory plots (Gora et al. 2025b). However, in that study, lightning frequency was acting as a proxy for storm activity, and the reported correlations thus implicitly include the effects of storm‐associated wind damage.

### 4.2 Sensitivity of AGB to Lightning Frequency and Compositional Acclimation

We used our model to assess the vulnerability of BCI to changes in lightning frequency (Price and Rind 1994; Banerjee et al. 2014; Finney et al. 2018; Charn and Parishani 2021). We found that the effects of increasing lightning frequency on AGB depended on our specific implementation of mortality risk: changes in lightning frequency resulted in much less variation in AGB using our species‐specific lightning risk model (SSLR) than in our community‐level risk model (CLLR). The reduced variation in the SSLR model occurred because a few species had increased AGB with increased *λ*, leading to compositional acclimation. Such a result is consistent with other studies that have shown that some species tolerate disturbances better than others, resulting in community reassembly (Ding et al. 2012; Cole et al. 2014; Seidl and Turner 2022). In the specific case of lightning, the limited information on species‐level resistance to lightning in tropical forests also indicates that some species are more sensitive than others (Richards et al. 2022). Consistent with observations, two large‐statured species ( *Dipteryx oleifera* and *Hura crepitans* ) were a relatively frequent target for lightning in our simulations because they tended to be tall; however, they were virtually insensitive to strikes. As a result, these two species drove most of the simulated AGB acclimation by lightning‐tolerant species.

We also found that while AGB generally declined in response to increasing lightning frequency, the frequency‐AGB curve showed a saturating response in our SSLR simulations. Again, compositional change drove this response. At high lightning frequencies, most large trees were from lightning‐tolerant species, and thus exhibit little sensitivity to lightning. Saturating responses to disturbance frequency have occasionally been observed elsewhere (Williams et al. 2013), and others have found that such responses can reflect acclimation or adaptation of a forest to disturbance and thus the severity of the disturbance (Thom et al. 2017; O'Connor et al. 2017). Our analysis of lightning disturbance should be distinguished from the case of stand‐replacing disturbance, which has shown stronger AGB sensitivity at higher disturbance frequencies (Pugh et al. 2019). Indeed, a lightning strike may result in no mortality if all impacted species are lightning‐tolerant.

Our results provoke hypotheses related to other sites. Spatially, lightning frequencies vary dramatically across forest and savanna ecosystems (Christian et al. 2003). While investigation of other sites is beyond the scope of this paper, our results suggest that the sites with low lightning frequency would be more sensitive to a change in lightning frequency than sites with high lightning frequency. We also expect that sensitivity to lightning will be impacted by forest structure, with more uniform canopies promoting more crown‐to‐crown flashover of lightning strikes and thus having higher mortality.

### 4.3 Implications for Further Empirical and Modeling Work

The analyses presented here suggest the need for further empirical and modeling work. First, although we found that our lightning‐enabled simulations were consistent with observations, the model error bars were much larger than the observational error bars. Reducing the size of the simulation uncertainty should thus be a priority. A next step would be a sensitivity analysis that apportions the output uncertainty to uncertainty in particular parameters (Saltelli 2002; Puy, Piano, et al. 2022). Then, the parameters driving the uncertainty could be targeted for additional measurements, or the model structure could be adjusted to better correspond to the current state of parameter knowledge. Another issue is that the lightning tolerance of many species is unknown, and these species were therefore treated as “neutral” in our analysis. If any of these species are in fact extremely lightning tolerant, the rate and magnitude of the compositional acclimation effect could increase substantially. Conversely, if a “neutral” species turns out to be lightning‐intolerant, then given the small effect sizes for species with $s_{i}>0.5$, our current results suggest that the overall implications of this change for AGB would be minimal.

Because our model formulation is species‐specific, individual‐based, and spatially explicit, it includes the most essential features related to lightning mortality. Nevertheless, it is also parsimonious, and does not include soil water, nutrients, or interannual variability in meteorological drivers. A recent version of TROLL has included soil water (Schmitt et al. 2025; Maréchaux et al. 2025), so TROLL can now be used to study soil water‐lightning interactions. Treatment of interannual climate variability should also be the subject of further study. While nutrients have not yet been included in any version of TROLL, the impact of nutrients on biomass and biomass growth in old‐growth forests on BCI is likely to be modest (Wright et al. 2018).

Our lightning‐enabled TROLL simulations suggest a number of insights and challenges specifically for future modeling studies. First, it is possible for a no‐lightning model to do a reasonably good job of simulating AGB, GPP, N60, and to a lesser extent, N10. But a no‐lightning model would not be able to capture the spatial variation of lightning across sites (Christian et al. 2003). Moreover, a no‐lightning model would also be biased at a single site if there are temporal trends in lightning frequency, although the bias may not be that large for AGB due to compositional acclimation. However, compositional and size‐related biases may be substantial. It remains unknown if the incorporation of lightning could reduce global model biases (Koch et al. 2021), but we encourage future research in that direction. Further, we note that changes in *λ* will not happen alone. Increases in lightning frequency are expected to be accompanied by increases in windthrow because both factors are produced by convective activity (Gora et al. 2025b). Furthermore, changes in atmospheric CO_2_ concentrations, temperature, rainfall, and other factors would also likely accompany changes in *λ* (IPCC 2021). Simulations of climate change, that include the full set of multiple interacting meteorological variables, should be conducted and analyzed. To the end of generating climate change simulations, close collaboration between atmospheric scientists and ecosystem modelers would help ensure realistic estimates of changes in lightning strike frequency.

This work also highlights the importance of species‐level models, rather than models based on plant functional types. Compositional acclimation to lightning was mainly driven by two species (i.e., < 10% of the species with some capacity to tolerate lightning), and connections between lightning tolerance and the functional traits that are usually used to parameterize models are relatively weak (Richards et al. 2022), indicating a need for species‐specific modeling. Beyond lightning, this work also suggests promise for the application of species‐level models to changes in other disturbance types such as droughts (da Costa et al. 2010).

## Author Contributions

**David Medvigy:** conceptualization, data curation, investigation, methodology, software, validation, writing – original draft, writing – review and editing. **Evan M. Gora:** conceptualization, funding acquisition, methodology, writing – review and editing. **Stephen P. Yanoviak:** conceptualization, funding acquisition, writing – review and editing.

## Funding

This work was supported by Smithsonian Tropical Research Institute, Tupper Postdoctoral Fellowship. National Science Foundation Graduate Research Fellowship Program, 2015188266. National Geographic Society, 9703‐15. Division of Environmental Biology, 1354060, 1655346, 2213245, 2213246, 2241507.

## Conflicts of Interest

The authors declare no conflicts of interest.

## Supporting information


**Data S1:** gcb70635‐sup‐0001‐Supinfo.pdf.

## Data Availability

The data and code that support the findings of this study are openly available in Zenodo at (https://doi.org/10.5281/zenodo.15722990).
