# Supplementary material for: Compositional Acclimation Can Lessen Tropical Forest Change in Response to Increasing Lightning Frequency: Insights From Simulation Modeling
Source: Glob Chang Biol. 2025 Dec 8;31(12):e70635. doi: 10.1111/gcb.70635 (PMC12683616; doi:10.1111/gcb.70635)
Supplement: Supplementary file 1 — Data S1: gcb70635‐sup‐0001‐Supinfo.pdf. [file GCB-31-e70635-s001.pdf]

Supplementary Materials for

Compositional acclimation can lessen tropical forest change in response to increasing lightning frequency: insights from simulation modeling

David Medvigy[1*], Evan M. Gora[2,3], Stephen P. Yanoviak[3,4]

[1]Department of Biological Sciences, University of Notre Dame, Notre Dame, IN 46556, USA

[2]Cary Institute of Ecosystem Studies, Millbrook, New York, NY, USA

[3]Smithsonian Tropical Research Institute, Balboa, Panama

[4]Department of Biology, University of Louisville, Louisville, KY, USA

*Correspondence: dmedvigy@nd.edu

The file includes:

Supplementary Material 1: Parameterization of the community level mortality risk model

Supplementary Material 2: Species Parameterization

Supplementary Material 3: Uncertainty Analysis

Supplementary Material 4: Transient response to a change in lightning frequency

Supplementary Material 5: Additional model evaluations

Supplementary Material 6: Parameter sets consistent with observations

Supplementary References

Supplementary Material 1: Parameterization of the community level mortality risk model

Qualitatively, the CLLR model (Eq. 1) was able to capture the basic pattern of observations regardless of the value of *d* (*d* indicates the fraction of lightning-damaged trees that eventually die from the strike) (Fig. S1). The largest biases occurred for bins with the fewest trees (large *D* and small *r*), which is consistent with our objective function which prioritizes bins with larger numbers of trees (Eq. 2). We then quantitatively examined our CLLR model fits for all values of *d* between 0 and 1 with a step of 0.05. Averaged over the 15 bins, the probability biases were always less than 0.05, root mean square probability errors were less than 0.09, and coefficients of determination were all larger than 0.86.

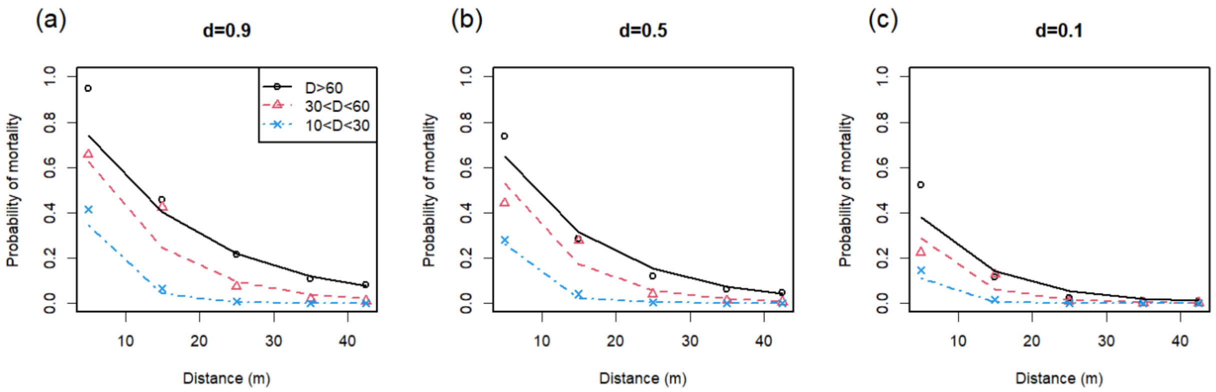

Figure S1: Validation of the two-parameter community-level lightning mortality risk model. In each panel, the results are aggregated by *D* (DBH in cm; denoted by different curves) and by distance from the strike (*x*-axis). The different panels represent different values of *d*: (a) *d*=0.9, (b) *d*=0.5, (c) *d*=0.1.

Supplementary Material 2: Species Parameterization

Species definitions are shown in Table S1. Leaf mass per unit area (LMA), leaf %N (Nmass), and leaf %P (Pmass) were obtained using public data in the TRY database (Kattge et al. 2020). Raw TRY downloads with citations to the original papers are provided in Medvigy et al. (2025). Wood specific gravity (wsg) and allometric parameters (dbhthresh, a, k, b) were taken from Martínez-Cano et al. (2019). The parameter governing the species-specific lightning effect (DLO; Eq. 3 in main text) was derived from Richards et al. (2022). PHYSIO is defined below.

Due to limitations on leaf %P, two different methods were used to compute leaf physiological parameters. The default TROLL model requires %P and uses it to calculate V_{cmax}, J_{max}, and R_{dark} (all on a mass basis) according to:

$$\log_{10}(V_{cmax}) = \min\left(-1.56 + 0.43\log_{10}(1000\,Nmass) + 0.37\log_{10}\left(\frac{10000}{LMA}\right), -0.80 + 0.45\log_{10}(1000\,Pmass) + 0.25\log_{10}\left(\frac{10000}{LMA}\right)\right)\quad \text{(Eq. S1a)}$$

$$\log_{10}(J_{max}) = \min\left(-1.50 + 0.41\log_{10}(1000\,Nmass) + 0.45\log_{10}\left(\frac{10000}{LMA}\right), -0.74 + 0.44\log_{10}(1000\,Pmass) + 0.32\log_{10}\left(\frac{10000}{LMA}\right)\right)\quad \text{(Eq. S1b)}$$

$$R_{dark} = 1.3893 + 0.0728\,Nmass * LMA + 0.0015\,Pmass * LMA + 0.0095 * V_{cmax}\,LMA - 0.0358 * 26.2 \quad \text{(Eq. 1c)}$$

Equations S1a and S1b come from Domingues et al. (2010), and Eq. S1c comes from Atkin et al. (2015). However, we did not have leaf %P for 7 of the 103 species. For these 7 species, we computed the physiological parameters according to:

$$\ln(V_{cmax}) = \frac{\left(1.993 + 2.555\ \ln(Nmass * LMA) - 0.372\ln\left(\frac{1}{LMA}\right) + 0.422\ln(Nmass * LMA)\ln(1/LMA)\right)}{LMA} \quad (\text{Eq. 2a})$$

$$\ln(J_{max}) = \frac{1.197 + 0.847\ \ln(V_{cmax}\ LMA)}{LMA} \quad (\text{Eq. 2b})$$

$$\log_{10}(R_{dark}) = -0.469 + 0.329\ \log_{10}(Nmass * LMA) + 0.204\log_{10}(LMA) \quad (\text{Eq. 2c})$$

Equations S2a and S2b come from Walker et al. (2014), and Eq. S2c again comes from Atkin et al. (2015).

Table S1: Species definitions. "LMA" is leaf mass per unit area. "Nmass" is leaf %N. "Pmass" is leaf %P. For species with PHYSIO=1, leaf %P is required and we used Equations S1a-c. For PHYSIO=0, leaf %P is not required and we used Equations S2a-c. "wsg" is the wood specific gravity. "dbhthresh" is a DBH threshold that is used to determine reproductive maturity and the DBH for which allocation to wood is reduced (Maréchaux and Chave 2017). "a", "k", and "b" allometric parameters that determine the DBH-Height relationship (Martínez-Cano et al. 2019). "DLO" is log-odds difference defined in Eq. 3 with data from Richards et al. (2022).

Species Name	LMA	Nmass	Pmass	PHYSIO	wsg	dbhthresh	a	k	b	Δ_i
dimensionless	**g/m2**	**g/g**	**g/g**	**dimensionless**	**g/cm3**	**m**	**m**	**m^b**	**dimensionless**	**dimensionless**
**Alchornea_costaricensis**	61.0	0.0250	0.00130	1	0.393	0.809	53.9	0.618	0.761	0.000
**Alseis_blackiana**	36.5	0.0323	0.00090	1	0.578	0.911	61.2	0.933	0.700	-0.365
**Amaioua_corymbosa**	62.1	0.0200	0.00117	1	0.678	0.138	60.1	0.880	0.709	0.000
**Anacardium_excelsum**	62.1	0.0164	0.00147	1	0.421	1.975	52.4	0.565	0.773	0.130
**Andira_inermis**	53.3	0.0301	0.00128	1	0.648	0.445	61.6	0.952	0.697	0.000

**Apeiba_membranacea**	50.1	0.0241	0.00150	1	0.321	1.146	54.4	0.636	0.756	0.839
**Apeiba_tibourbou**	50.9	0.0252	0.00101	1	0.343	0.386	44.9	0.361	0.835	0.000
**Astronium_graveolens**	61.4	0.0271	0.00155	1	0.808	0.855	57.3	0.750	0.732	0.000
**Beilschmiedia_tovarensis**	92.7	0.0166	0.00110	1	0.528	0.955	59.4	0.844	0.715	-0.340
**Brosimum_alicastrum**	71.7	0.0229	0.00088	1	0.650	1.294	58.8	0.815	0.720	0.000
**Brosimum_guianense**	57.8	0.0226	0.00094	1	0.748	0.510	58.8	0.816	0.720	0.000
**Calophyllum_longifolium**	130.1	0.0111	0.00068	1	0.541	1.200	57.8	0.774	0.728	0.000
**Casearia_arborea**	40.9	0.0282	0.00098	1	0.562	0.578	56.2	0.706	0.741	0.000
**Casearia_sylvestris**	66.7	0.0247	0.00074	1	0.662	0.264	58.3	0.795	0.724	0.000
**Cecropia_insignis**	65.0	0.0266	0.00143	1	0.462	1.089	44.9	0.362	0.835	0.000
**Cecropia_obtusifolia**	60.5	0.0267	0.00177	1	0.305	0.554	42.1	0.302	0.859	0.000
**Ceiba_pentandra**	78.4	0.0235	0.00167	1	0.305	2.300	48.0	0.436	0.810	0.000
**Celtis_schippii**	54.9	0.0315	0.00189	1	0.579	0.385	57.8	0.774	0.728	0.000
**Chrysophyllum_argenteum**	76.2	0.0237	0.00064	1	0.736	0.548	59.9	0.870	0.711	0.000
**Chrysophyllum_cainito**	77.4	0.0182	0.00102	1	0.777	0.947	60.7	0.910	0.704	0.000
**Cordia_alliodora**	44.1	0.0287	0.00169	1	0.434	0.642	54.0	0.621	0.760	0.000
**Cordia_bicolor**	51.8	0.0246	0.00088	1	0.373	0.470	56.4	0.714	0.740	0.303
**Damburneya_umbrosa**	84.9	0.0162	0.00078	1	0.513	0.254	54.8	0.651	0.753	0.000
**Dendropanax_arboreus**	67.9	0.0174	0.00102	1	0.411	0.730	57.5	0.757	0.731	0.000
**Desmopsis_panamensis**	56.1	0.0232	0.00116	1	0.531	0.131	59.7	0.857	0.713	-0.610
**Dipteryx_oleifera**	55.3	0.0244	0.00131	1	0.867	1.800	59.8	0.864	0.712	-99999999.000
**Enterolobium_schomburgkii**	132.6	0.0288	0.00117	1	0.636	0.331	54.9	0.654	0.752	0.000
**Faramea_occidentalis**	62.7	0.0189	0.00070	1	0.609	0.222	57.8	0.770	0.729	0.369
**Ficus_maxima**	58.0	0.0251	0.00126	1	0.373	0.448	51.3	0.530	0.783	0.000
**Ficus_yoponensis**	91.8	0.0193	0.00130	1	0.342	0.927	44.0	0.342	0.842	0.000
**Garcinia_madruno**	95.7	0.0131	0.00048	1	0.668	0.300	58.4	0.798	0.723	0.000
**Garcinia_recondita**	105.6	0.0112	0.00067	1	0.654	0.272	59.6	0.852	0.714	-0.727
**Genipa_americana**	52.2	0.0172	0.00050	1	0.681	0.508	63.1	1.036	0.684	0.000
**Guarea_bullata**	70.8	0.0203	0.00140	1	0.668	0.215	61.2	0.935	0.700	0.000
**Guarea_grandifolia**	55.2	0.0273	0.00184	1	0.579	1.090	58.8	0.818	0.720	0.000
**Guarea_guidonia**	61.0	0.0272	0.00217	1	0.560	0.546	59.5	0.850	0.714	0.000
**Guazuma_ulmifolia**	43.4	0.0246	0.00072	1	0.495	0.970	51.5	0.537	0.781	0.000
**Gustavia_superba**	49.5	0.0342	0.00200	1	0.612	0.441	56.0	0.697	0.743	-2.026
**Handroanthus_guayacan**	52.2	0.0353	0.00120	1	0.790	1.699	60.2	0.881	0.709	0.000
**Hasseltia_floribunda**	47.4	0.0287	0.00179	1	0.565	0.268	58.9	0.822	0.719	0.000
**Heisteria_acuminata**	69.9	0.0159	0.00061	1	0.682	0.143	57.7	0.765	0.730	0.000
**Hieronyma_alchorneoides**	52.3	0.0230	0.00158	1	0.585	1.115	60.4	0.891	0.707	0.000
**Hirtella_triandra**	55.7	0.0168	0.00063	1	0.623	0.415	58.8	0.815	0.720	-0.992

**Hura_crepitans**	57.6	0.0314	0.00370	1	0.370	2.340	63.0	1.004	0.680	-100000000.000
**Inga_acuminata**	46.2	0.0310	0.00120	1	0.321	0.256	54.5	0.641	0.755	0.000
**Inga_marginata**	60.1	0.0302	0.00172	1	0.474	0.598	55.7	0.686	0.746	0.000
**Inga_sapindoides**	49.4	0.0384	0.00157	1	0.612	0.314	58.3	0.793	0.724	0.000
**Inga_spectabilis**	84.8	0.0297	0.00123	1	0.501	0.390	55.0	0.657	0.752	0.000
**Inga_umbellifera**	55.7	0.0317	0.00101	1	0.665	0.124	59.5	0.849	0.714	0.000
**Jacaranda_copaia**	63.1	0.0263	0.00091	1	0.374	1.373	52.9	0.583	0.769	0.142
**Lacistema_aggregatum**	50.5	0.0228	0.00153	1	0.558	0.210	60.1	0.879	0.709	0.000
**Lacmellea_panamensis**	59.9	0.0193	0.00080	1	0.502	0.494	52.7	0.576	0.771	0.000
**Laetia_procera**	70.6	0.0248	0.00113	1	0.579	0.622	49.8	0.486	0.795	0.000
**Lonchocarpus_heptaphyllus**	51.3	0.0246	0.00106	1	0.666	0.570	59.1	0.830	0.718	0.000
**Luehea_seemannii**	61.7	0.0215	0.00133	1	0.573	1.149	54.0	0.621	0.760	0.000
**Maquira_guianensis**	58.3	0.0217	0.00058	1	0.643	0.189	58.3	0.792	0.725	0.000
**Marila_laxiflora**	45.5	0.0186	0.00087	1	0.673	0.051	57.2	0.748	0.733	0.000
**Miconia_argentea**	74.9	0.0179	0.00048	1	0.697	0.357	52.1	0.556	0.776	0.000
**Mouriri_myrtilloides**	76.8	0.0226	0.00009	1	0.788	0.197	60.4	0.895	0.706	0.000
**Myrcia_splendens**	76.8	0.0148	0.00059	1	0.659	0.146	58.0	0.779	0.727	0.000
**Myrospermum_frutescens**	50.2	0.0290	0.00016	1	0.875	0.585	62.2	0.987	0.692	0.000
**Ochroma_pyramidale**	86.5	0.0233	0.00142	1	0.191	0.705	35.4	0.194	0.914	0.000
**Ocotea_puberula**	59.5	0.0187	0.00040	1	0.389	0.719	56.6	0.720	0.739	0.000
**Ocotea_whitei**	87.6	0.0168	Na	0	0.426	0.863	56.4	0.711	0.740	-0.258
**Ormosia_macrocalyx**	55.6	0.0250	0.00310	1	0.650	0.749	59.4	0.846	0.715	0.000
**Perebea_xanthochyma**	52.8	0.0161	0.00104	1	0.623	0.218	58.4	0.797	0.724	0.000
**Platymiscium_pinnatum**	57.8	0.0351	0.00260	1	0.761	0.830	60.0	0.871	0.710	0.000
**Pombalia_prunifolia**	39.5	0.0223	Na	0	0.583	0.084	60.7	0.910	0.704	0.162
**Poulsenia_armata**	73.1	0.0175	0.00164	1	0.315	0.968	55.5	0.676	0.748	0.000
**Pourouma_bicolor**	62.1	0.0202	0.00098	1	0.357	0.530	53.7	0.612	0.762	0.000
**Pouteria_reticulata**	64.4	0.0262	0.00084	1	0.694	0.865	60.7	0.905	0.705	-99999999.000
**Prioria_copaifera**	93.8	0.0207	Na	0	0.432	1.405	59.9	0.870	0.711	0.151
**Protium_confusum**	52.2	0.0199	0.00090	1	0.536	0.190	53.2	0.593	0.767	0.000
**Protium_panamense**	73.6	0.0181	0.00112	1	0.438	0.344	60.1	0.879	0.709	0.000
**Protium_tenuifolium**	69.4	0.0186	0.00082	1	0.586	0.553	58.6	0.808	0.722	0.296
**Pseudobombax_septenatum**	47.8	0.0197	0.00166	1	0.296	1.305	52.3	0.563	0.774	0.000
**Pterocarpus_hayesii**	33.4	0.0311	0.00137	1	0.511	0.720	59.8	0.865	0.711	0.000
**Quararibea_asterolepis**	61.9	0.0239	Na	0	0.468	1.308	60.0	0.872	0.710	0.125
**Sapium_glandulosum**	67.9	0.0230	0.00183	1	0.502	0.777	55.0	0.658	0.752	0.000
**Simarouba_amara**	61.2	0.0178	0.00067	1	0.390	0.789	55.5	0.677	0.747	-0.133
**Sloanea_terniflora**	64.0	0.0161	0.00049	1	0.772	1.247	62.6	1.008	0.689	0.000
**Spondias_mombin**	59.7	0.0225	0.00116	1	0.423	0.881	51.5	0.536	0.781	0.000

**Spondias_radlkoferi**	54.1	0.0165	0.00116	1	0.366	0.948	57.4	0.753	0.732	0.000
**Sterculia_apetala**	62.5	0.0234	0.00091	1	0.439	2.080	56.9	0.735	0.735	0.000
**Symphonia_globulifera**	98.5	0.0173	0.00088	1	0.545	0.752	57.4	0.754	0.732	0.000
**Tabebuia_rosea**	50.0	0.0187	0.00099	1	0.482	1.089	58.9	0.820	0.719	0.719
**Tabernaemontana_arborea**	44.0	0.0314	Na	0	0.538	0.856	59.1	0.832	0.717	0.650
**Tachigali_panamensis**	57.6	0.0282	Na	0	0.578	0.874	58.8	0.816	0.720	-0.442
**Terminalia_amazonia**	62.0	0.0177	0.00129	1	0.665	0.911	57.9	0.777	0.727	0.000
**Terminalia_oblonga**	40.4	0.0248	0.00188	1	0.634	0.739	56.1	0.703	0.742	0.000
**Tetragastris_panamensis**	86.7	0.0154	0.00060	1	0.589	0.766	59.7	0.858	0.713	-0.095
**Trattinnickia_aspera**	64.1	0.0193	0.00056	1	0.436	0.829	51.1	0.524	0.784	0.000
**Trema_micrantha**	50.4	0.0283	0.00140	1	0.386	0.640	39.1	0.248	0.884	0.000
**Trichilia_pallida**	49.7	0.0269	0.00188	1	0.573	0.238	58.3	0.792	0.725	0.000
**Trichilia_tuberculata**	70.7	0.0231	Na	0	0.658	0.653	60.3	0.889	0.708	0.050
**Turpinia_occidentalis**	46.6	0.0254	0.00113	1	0.407	0.554	51.6	0.539	0.780	0.000
**Unonopsis_pittieri**	58.1	0.0203	0.00083	1	0.428	0.304	57.5	0.759	0.731	0.000
**Virola_multiflora**	83.4	0.0174	0.00060	1	0.448	0.650	54.4	0.636	0.757	0.000
**Virola_nobilis**	66.5	0.0187	0.00088	1	0.407	0.984	57.0	0.737	0.735	0.246
**Virola_sebifera**	69.4	0.0237	0.00092	1	0.478	0.490	58.2	0.789	0.725	0.000
**Vochysia_ferruginea**	64.7	0.0172	0.00091	1	0.389	0.742	51.2	0.529	0.783	0.000
**Zanthoxylum_ekmanii**	63.9	0.0283	0.00171	1	0.435	0.696	44.5	0.351	0.839	0.000
**Zanthoxylum_panamense**	51.8	0.0286	0.00116	1	0.552	0.535	58.4	0.798	0.723	0.000

Supplementary Material 3: Uncertainty Analysis

The parameters subjected to uncertainty analysis and their allowed ranges are shown in Table 1 of the main text. The range for ϕ was similar to Schmitt et al. (2023). We allowed m_0 to take on smaller values than Schmitt et al. (2023) because we are effectively taking the process of lightning out of baseline mortality and representing it explicitly in CLLR TROLL and SSLR TROLL. We let f_{below} be uniformly distributed on (0.1, 0.5), forcing the sum $f_{\text{wood}} + f_{\text{canopy}}$ to be uniformly distributed on (0.5, 0.9). We took θ to be uniformly distributed on $\left(\frac{\pi}{12}, \frac{5\pi}{12}\right)$. This procedure is advantageous because it maps the ratio $f_{\text{canopy}}/f_{\text{wood}}$, with support on $(0,\infty)$, to θ, which has support over the finite range $(0,\pi/2)$. For CLLR TROLL and SSLR TROLL, we additionally analyzed uncertainty arising from λ and d (Table 1). λ was taken to be uniformly distributed over its observed 95% confidence interval (i.e., 10.9-14.5 CG fl km^{-2} yr^{-1}) and d was taken to be uniformly distributed over the full possible range of values (0,1).

We did not include the crown radius allocation parameters in our uncertainty analysis because we had access to a local allometry (Martínez Cano et al. 2019). However, we did have to convert the original equation for crown area (CA) to an equation for crown radius (CR). The original equation was written as

$$CA = 0.66 D_{cm}^{1.34} \quad ,$$

with D_{cm} representing diameter at breast height measured in cm. Using $CA = \pi CR^2$ and $D = 0.01 D_{cm}$, the allometric equation was written as

$$CR = 10 D^{0.67} \quad .$$

We used a Sobol' sequence based on quasi-random number to efficiently sample these multi-dimensional parameter spaces (Kucherenko et al. 2015) using the R package "sensobol" (Puy et al. 2022). We set the sample size of the base sample matrix to 2^{13}, yielding 49,152

parameter sets for the no-lightning model formulation (4 free parameters) and 65,536 parameter sets for the model formulations with lightning (6 free parameters). Because mortality and recruitment are stochastic processes in TROLL, an ensemble of simulations is potentially necessary for each parameter set. In a preliminary analysis, we evaluated how bootstrapped confidence intervals on Sobol' indices (Puy et al. 2020) varied with both the sample size of the base Sobol' matrix and the number of ensemble members. We found that it was far more computationally efficient to increase the sample size of the base Sobol' matrix rather than run multiple realizations with the same parameter set. Therefore, we simulated only one realization for each parameter set.

Supplementary Material 4: Transient response to a change in lightning frequency

We simulated additional 600-year ensembles using our filtered parameter sets. The first 500 years were forced with present-day lightning frequency ($\lambda_{current}$). Over the next 100 years, lightning frequency varied linearly in simulation time (*t*; years), reaching a final value ($\lambda_{transient,i}$) at year 600:

$$\lambda(t) = \lambda_{current} + (\lambda_{transient,i} - \lambda_{current})\,(t-500)/100 \qquad (500 <= t <= 600)$$

We simulated these ensembles for different values of $\lambda_{transient,i}$ ranging from 9 to 23 CG strikes km^{-2} yr^{-1}. We denote the ensemble associated with $\lambda_{transient,i}$ as $E_{transient,i}$. To assess the degree of acclimation, these transient simulations were compared to our original 600 year simulations that were forced with fixed λ. We denote these ensembles by $E_{fixed,i}$. The ensemble with a fixed value of λ equal to $\lambda_{current}$ was denoted $E_{fixed,current}$.

One of the largest effects of lightning that we simulated was on species composition (Figs. 5, 6). As an index of composition, we computed the ratio of lightning-tolerant species AGB to total AGB, and we denote this ratio by TolFrac. To facilitate comparison of multiple ensembles and assess the degree of acclimation, we applied a linear transformation on TolFrac. In particular, we wanted the transformed variable, TolFracTrans, to have the following properties:

1. TolFracTrans = 0 if TolFrac from $E_{transient,i}$ is equal to the TolFrac from $E_{fixed,current}$ at the same time point. This case indicates no acclimation.
2. TolFracTrans = 1 if TolFrac from $E_{transient,i}$ is equal to the TolFrac from $E_{fixed,i}$ at the same time point. This case indicates complete acclimation to $\lambda_{transient,i}$.

Applying a linear transformation subject to these two properties yields:

$$TolFracTrans_i = (TolFrac_i - TolFrac_{fixed,current}) / (TolFrac_{fixed,i} - TolFrac_{fixed,current})$$

The results are shown in Fig. S2. The salient points are that: (i) $TolFracTrans_i > 0$, so compositional acclimation is occurring. (ii) The system is generally 20-30% acclimated when the transient ends. This incomplete acclimation was expected because changes in species composition are strongly controlled by the overall turnover rate and thus change gradually over time as mortalities occur. (iii) the final values of $TolFracTrans_i$ do not appear to be dependent on $\lambda_{transient,i}$. This result is reflective of the fact that other processes contribute to mortality and thus turnover.

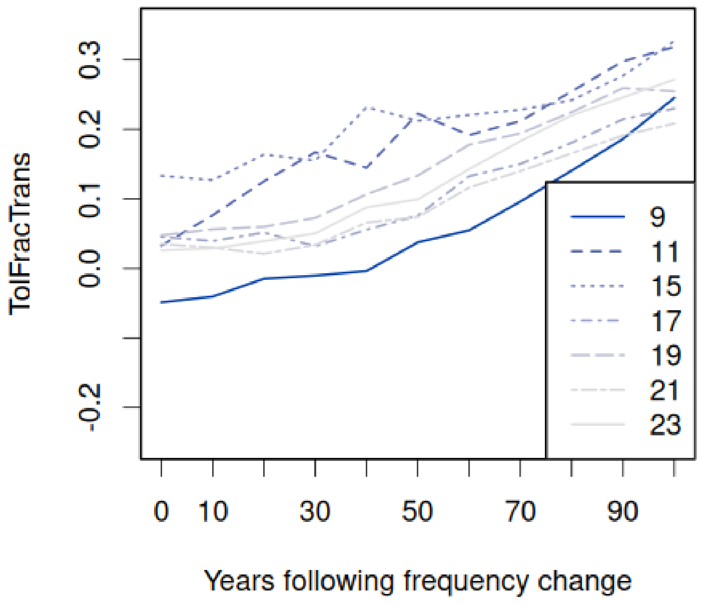

Figure S2: Transient response to a change in lightning frequencies. The figure shows the final 100 years of 600 year simulations. All simulations were run for 500 years with the same lightning frequency of 13 CG strikes km^{-2} yr^{-1}. Lightning frequency then linearly varied until it reached a target value at the end of the simulation. Target values ranged from 9-23 CG strikes km^{-2} yr^{-1}, as indicated in the legend.

Supplementary Material 5: Additional model evaluations

Our primary model evaluation is given in Fig. 2 of the main text. Here, we also evaluate the model's ability to simulate the number of trees with diameter at breast height larger than 30cm. Different versions of TROLL (no lightning, CLLR lightning, SSLR lightning) are compared to each other and the observations (Fig. S3). There is little difference across the model versions. The observed range falls within the upper half of simulated values.

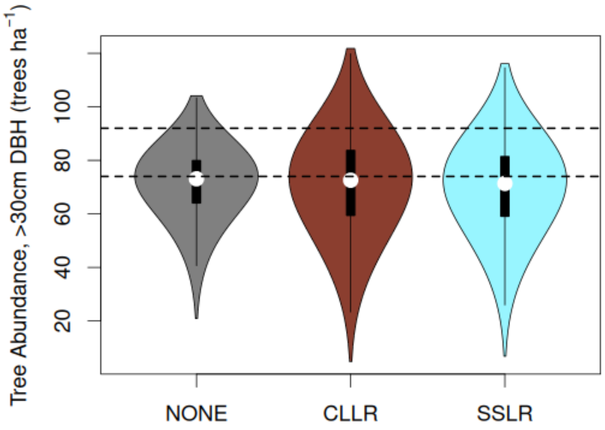

Figure S3: TROLL model evaluation of the number of trees with DBH greater than 30cm (N30) for different implementations of lightning. The dashed black lines indicate the 95% confidence interval on the observations. The "NONE" distributions correspond the no-lightning model, the "CCLR" distributions correspond to TROLL with a community-level lightning risk model, and the "SSLR" distributions correspond to TROLL with a species-specific lightning risk model.

We also evaluated the model's ability to simulate the number of trees killed per strike. The model simulated 1.2 trees (DBH>60cm) killed per strike under current-day lightning

frequencies (Fig. 7b in the main text). This result is consistent with the observations of Yanoviak et al. (2020), who estimated 0.94 (range: 0-4) trees (DBH>60cm) killed per strike within 13 months, and a further 2.13 trees (>60cm DBH) that were alive but damaged. It is not known whether these damaged trees have now died or will die prematurely as a result of the strike. Because TROLL accounts for a certain fraction of damaged trees eventually dying (through the *d* parameter), it is unsurprising that the simulated number of kills per strike is a bit higher than the observed best estimate. For trees with DBH>10cm, we simulated 5.1 kills/strike on average. Yanoviak et al. (2020) reported 3.5 killed/strike and 11.4 damaged/strike. Thus, our results would be equivalent to the mean numbers of Yanoviak et al. (2020) if 14% of damaged trees eventually died due to the strike, which seems plausible (Gora et al. 2025b).

Supplementary Material 6: Parameter sets consistent with observations

The different versions of TROLL (no lightning, CLLR lightning, SSLR lightning) required different parameter sets in order to be consistent with observations (Fig. S4). In particular, the baseline mortality parameter was much higher in no-lightning simulations than in CLLR or SSLR.

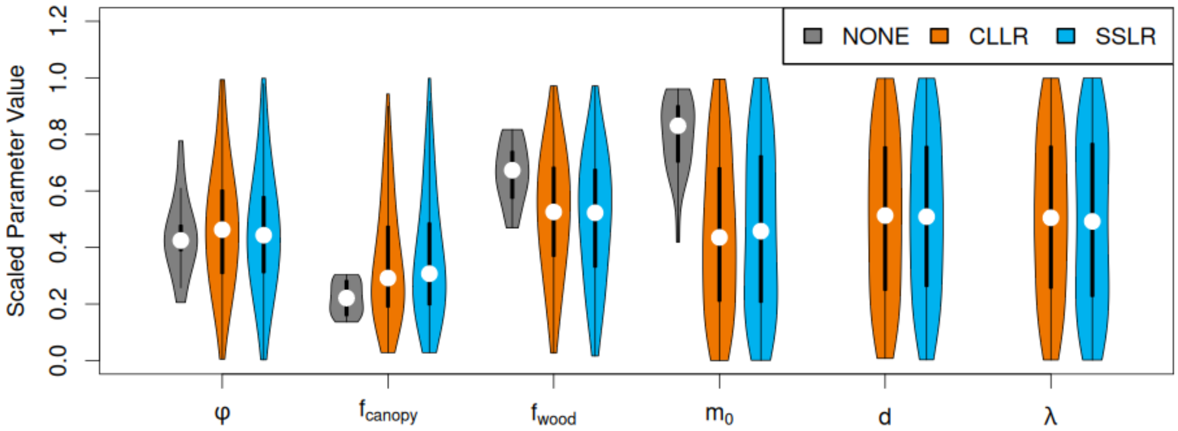

Figure S4: Univariate parameter distributions for parameter sets that were consistent with observed AGB, GPP, N10, and N60. All parameter values were linearly scaled to facilitate comparison. A scaled value of 0 represents the minimum parameter value and a scaled value of 1 represents the maximum parameter value (the maximum and minimum values are given in Table 1). Results are shown for no-lightning TROLL (NONE), community-level lightning risk TROLL (CLLR), and species-specific lightning risk TROLL (SSLR).

Supplementary References

Atkin, O.K., Bloomfield, K.J., Reich, P.B., Tjoelker, M.G., Asner, G.P., Bonal, D., Bönisch, G., Bradford, M.G., Cernusak, L.A., Cosio, E.G. and Creek, D., 2015. Global variability in leaf respiration in relation to climate, plant functional types and leaf traits. *New Phytologist*, *206*(2), pp.614-636.

Domingues, T.F., Meir, P., Feldpausch, T.R., Saiz, G., Veenendaal, E.M., Schrodt, F., Bird, M., Djagbletey, G., Hien, F., Compaore, H. and Diallo, A., 2010. Co-limitation of photosynthetic capacity by nitrogen and phosphorus in West Africa woodlands. *Plant, Cell & Environment*, *33*(6), pp.959-980.

Gora, E. M., H. C. Muller-Landau, K. C. Cushman, J. H. Richards, P. M. Bitzer, J. C. Burchfield, P. Narváez, and S. P. Yanoviak. 2025b. How some tropical trees benefit from being struck by lightning: evidence for Dipteryx oleifera and other large-statured trees. New Phytologist 246:1554–1566.

Kattge, J, Boenisch, G, Diaz, S, et al. TRY plant trait database - enhanced coverage and open access. Glob Change Biol. 2020; 26: 119-188. https://doi.org/10.1111/gcb.14904.

Kucherenko S, Albrecht D, Saltelli A (2015). "Exploring Multi-Dimensional Spaces: A Comparison of Latin Hypercube and Quasi Monte Carlo Sampling Techniques." arXiv:1505.02350 [stat.AP], URL https://arxiv.org/abs/1505.02350

Maréchaux, I. and Chave, J., 2017. An individual-based forest model to jointly simulate carbon and tree diversity in Amazonia: description and applications. *Ecological Monographs*, *87*(4), pp.632-664.

Martínez Cano, I., Muller-Landau, H.C., Wright, S.J., Bohlman, S.A. and Pacala, S.W., 2019. Tropical tree height and crown allometries for the Barro Colorado Nature Monument, Panama: a comparison of alternative hierarchical models incorporating interspecific variation in relation to life history traits. *Biogeosciences*, *16*(4), pp.847-862.

Medvigy, D., E.M. Gora, and S.P. Yanoviak. (2025). davidmedvigy/rcontroll/lightning: v0.1.0. *Zenodo*. https://doi.org/10.5281/zenodo.15722990.

Puy, A., Piano, S.L., Saltelli, A. and Levin, S.A., 2022. Sensobol: An R package to compute variance-based sensitivity indices. *Journal of Statistical Software, 102*, pp.1-37.

Richards, J.H., Gora, E.M., Gutierrez, C., Burchfield, J.C., Bitzer, P.M. and Yanoviak, S.P., 2022. Tropical tree species differ in damage and mortality from lightning. *Nature Plants*, *8*(9), pp.1007-1013.

Schmitt, S., Salzet, G., Fischer, F.J., Maréchaux, I. and Chave, J., 2023. rcontroll: An R interface for the individual-based forest dynamics simulator TROLL. *Methods in Ecology and Evolution*, *14*(11), pp.2749-2757.

Walker, A.P., Beckerman, A.P., Gu, L., Kattge, J., Cernusak, L.A., Domingues, T.F., Scales, J.C., Wohlfahrt, G., Wullschleger, S.D. and Woodward, F.I., 2014. The relationship of leaf photosynthetic traits–Vcmax and Jmax–to leaf nitrogen, leaf phosphorus, and specific leaf area: a meta-analysis and modeling study. *Ecology and evolution*, *4*(16), pp.3218-3235.
